# Sex-specific associations of urinary mixed-metal concentrations with femoral bone mineral density among older people: an NHANES (2017–2020) analysis

**DOI:** 10.3389/fpubh.2024.1363362

**Published:** 2024-05-17

**Authors:** Hecheng Li, Guoliang Li, Mushi Yi, Jiazhen Zhou, Yaotang Deng, Yiqi Huang, Shuirong He, Xiaojing Meng, Lili Liu

**Affiliations:** ^1^School of Public Health, Southern Medical University, Guangzhou, China; ^2^Department of Toxicology, Guangdong Province Hospital for Occupational Disease Prevention and Treatment, Guangzhou, China; ^3^School of Public Health, Guangzhou Medical University, Guangzhou, China; ^4^School of Public Health, Sun Yat-sen University, Guangzhou, China

**Keywords:** urinary metal mixture, bone mineral density, NHANES, middle-aged and older population, WQS regression, BKMR

## Abstract

**Background:**

Heavy metal exposure is an important cause of reduced bone mineral density (BMD). Epidemiological studies focusing on the effects of mixed heavy metal exposure on BMD in middle-aged and older people are scarce. In single-metal studies, men and women have shown distinct responses of BMD to environmental metal exposure. This study therefore aimed to elucidate the association between mixed heavy metal exposure and BMD and to investigate whether it is sex-specific.

**Methods:**

Data from the 2017–2020 National Health and Nutrition Examination Survey were selected for this cross-sectional study. The study used three statistical methods, i.e., linear regression, Bayesian kernel machine regression (BKMR) modeling, and weighted quartiles (WQS) regression, to explore the association between the urinary concentrations of 11 metals (barium, cadmium, cobalt, cesium, manganese, molybdenum, lead, antimony, tin, thallium, and Tungsten), either individually or as a mixture, and total femoral BMD.

**Results:**

A total of 1,031 participants were included in this study. Femoral BMD was found to be higher in men than women. A significant negative correlation between the urinary concentrations of the 10 metals and femoral BMD was found in the overall cohort. Further gender sub-stratified analyses showed that in men, urinary metal concentrations were negatively correlated with femoral BMD, with cobalt and barium playing a significant and non-linear role in this effect. In women, although urinary metal concentrations negatively modulated femoral BMD, none of the correlations was statistically significant. Antimony showed sex-specific differences in its effect.

**Conclusion:**

The urinary concentrations of 10 mixed heavy metals were negatively correlated with femoral BMD in middle-aged and older participants, and this effect showed gender differences. These findings emphasize the differing role of mixed metal exposure in the process of BMD reduction between the sexes but require further validation by prospective studies.

## Introduction

Osteoporosis, a chronic systemic skeletal disease characterized by an insidious decrease in bone mineral density (BMD) and increased bone fragility, has become a widespread public health problem (1, 2). Bone loss affects 40 million people in the U.S. and is largely concentrated in the middle-aged and older population, severely impacting people’s quality of life (3, 4). Studies have shown that BMD declines significantly in people over 50 years of age, and prevalence rates are projected to continue to rise in the coming decades, adding a significant healthcare burden to society, with the cost of treating osteoporosis and fracture-related diseases expected to reach $25 billion by 2025 (5, 6). BMD adequately reflects bone strength and is a commonly used indicator for assessing bone health status, with femoral BMD being the most widely used in epidemiological studies (7, 8). Identifying risk factors for decreased BMD is an important measure to prevent the development of osteoporosis.

Heavy metals are prevalent in a variety of environmental media and can enter the body in a multitude of ways, including through food, drinking water, smoking, and occupational exposure (9). Exposure to heavy metals is known to be a risk factor for chronic bone diseases such as fractures and osteoporosis (10, 11). *In vitro* and *in vivo* studies have found that metal exposure affects bone tissue through distinct genetic, nutritional, and metabolic mechanisms (12, 13). For example, cadmium (Cd) can contribute to an increased risk of fractures and osteoporosis by promoting bone marrow mesenchymal stem cell (BMSC) senescence and mitochondrial dysfunction (14, 15). Environmental lead (Pb) is absorbed into the body through a variety of pathways, with bone trabeculae and cortex being the main targets for Pb accumulation. BMD decreases with the accumulation of Pb in the body, which in turn leads to the development of bone-related diseases (16). Chronic manganese (Mn) exposure was found to increase the risk of osteoporosis in a cohort of retired older adults, and this effect was particularly significant in women (17). However, because humans are inevitably exposed to multiple metals at the same time, exposure to any single metal cannot fully account for the onset and progression of disease; both synergistic and antagonistic interactions between metals play a role. Therefore, it is crucial to explore the effects of mixed exposure to multiple metals on BMD. Noticeably, sex-specific associations of heavy metals with liver fibrosis, kidney function and cognitive function were observed in previous studies (18–20).

Therefore, the present study, based on the most recent data from the National Health and Nutrition Examination Survey (NHANES), investigated the effects of the urinary concentrations of 11 metals, individually and in combination, on femoral BMD in the middle-aged and older adult population, and the potential gender specificity of this effect.

## Methods

### Study subjects

NHANES is a series of cross-sectional studies conducted by the U.S. Centers for Disease Control and Prevention to assess the health status of the national non-institutionalized U.S. population. The data are released following a two-year cycle, but because 2019–2020 was affected by the prevalence of coronavirus disease 2019, the 2017–2020 data were combined into one nationally representative dataset in this study, with a total of 15,560 individuals included. Initially, we excluded persons with missing urinary metal data (*N* = 11,624). Then, we excluded people ≤50 years of age and those with missing total femoral BMD data (*N* = 12,015). Finally, participants with incomplete baseline information were excluded (*N* = 8,751), leaving 1,031 participants with data for final analysis. All participants provided written informed consent to take part in the study. The National Ethical Review Board for Health Statistics Research approved the investigation protocol.

### Covariates

The main factors in the NHANES 2017–2020 database that may affect BMD were considered as covariates for inclusion in this study. The covariates primarily included age (years), sex (male or female), race/ethnicity (Mexican-American, other Hispanic, non-Hispanic White, non-Hispanic Black, or other), education level (less than 9th grade, grades 9–11, high school graduate, some college, or college graduate), marital status (married/cohabiting, widowed/divorced/separated, or never married), and intensity of work (strenuous, moderate, or none). Alcohol consumption status was categorized as never or ever based on ever having been exposed to alcoholic beverages. Smoking status was categorized as yes or no on the basis of tobacco use equivalent to at least 100 cigarettes in life. Body mass index (BMI) was categorized as <25, 25–30, or > 30 kg/m^2^. Diabetes mellitus was defined according to previous diagnosis (yes, borderline, or no). Hypertension was defined according to previous diagnosis (yes or no). Household economic status was categorized as ≤1.30, 1.30–1.85, or ≥ 1.85 based on the monthly household poverty level index, these categories were chosen because they represented commonly used percentages of the poverty guidelines (i.e., 130 percent and 185 percent of the guidelines), by federal programs, in determining eligibility.

### BMD testing

Total femoral BMD was estimated by dual-energy X-ray absorptiometry (DXA) (21). The BMD measurements were performed by professionally trained and certified radiologists. The femur scans provide bone measurements for the total femur. Participants who self-reported using contrast media in the past 7 days, were pregnant, or weighed more than 450 pounds were not allowed to participate in the DXA examination. Detailed information is recorded in the NHANES database.

### Exposure assessment

Data on the detection of 11 metals in urine were obtained from NHANES 2017–2020. Initial determinations of Ba, Cd, cobalt (Co), Cs, Mn, Mo, Pb, antimony (Sb), tin (Sn), thallium (Tl), and Tungsten (W) were made in spot urine using inductively coupled plasma mass spectrometry. Due to the different detection methods used in 2017–2018 and 2019–2020, the higher value of those given by the two methods was officially adopted as the limit of detection (LOD) for each metal to make the combined datasets compatible. The LOD divided by the square root of two was used to replace values below the LOD according to the NHANES standard. The percentage of persons below LOD for each metal is shown in the Supplementary Table S1. For >50% below LOD (Mn) also investigated as binary variables, coded 0 when below the LOD and 1 when above the LOD (22). To reduce the effect of urine dilution, the metal concentrations were corrected for urinary creatinine and expressed as micrograms per gram of creatinine.

### Statistical analysis

Data on the participants’ baseline characteristics are expressed as the mean (standard deviation) for continuous variables and frequency (percentage) for categorical variables. Differences between groups were compared using Student’s *t*-test or Kruskal-Wallis H test for continuous variables, and the χ^2^ test for categorical variables. Raw data for the urinary metal concentrations are presented as medians (upper quartile, lower quartile), and the logarithms of the urinary metal data were used in the statistical and analytical processes. Pearson correlation analysis was used to determine the correlation between the concentrations of the 10 urinary metals and the total BMD. The correlation between each urinary metal concentration and total BMD was assessed by weighted multiple linear regression modeling, incorporating different covariates for adjustment (model 1: unadjusted; model 2: adjusted for age, race, marital status, and BMI; model 3: adjusted for all covariates). Weighted quartiles (WQS) regression was used to assess the effect of multiple urinary metal concentrations on total BMD, with the contribution to the overall effect calculated through the corresponding weighted response for each metal. The WQS regression type was set to linear, and the data were randomly divided into training and validation datasets at a ratio of 4:6, with the number of replication results for analysis specified as 1,000. The Bayesian kernel machine regression (BKMR) model was used to assess the interaction effects and non-linear correlations of exposure to metals with respect to the study outcomes, after adjusting for potential confounders. The effects of all metals on total BMD were predicted by calculating the specific percentile exposure levels compared with the median exposure levels. All analyses were further stratified by gender and adjusted to include all covariates except gender. The analyses were performed using the statistical software packages SPSS 21.0 and R software version 4.3.1. *p*-values <0.05 were considered as statistically significant.

## Results

### Characteristics of participants

Table 1 shows the basic characteristics of the participants, stratified by sex. A total of 1,031 older people were included in this study, including 556 men and 475 women. The average age of the men was 64.43 years and that of the women was 63.53 years. The differences between men and women in marital status, BMI, smoking, alcohol consumption, diabetes mellitus, and intensity of work were statistically significant (*p* < 0.05). A greater proportion of men are physically overweight, while women are predominantly obese. The higher prevalence of diabetes mellitus in men than in women may be related to the higher frequency of unhealthy behaviors, such as smoking and alcohol consumption, among men. The mean femoral BMD was higher in men than women, at 0.998 g/cm^2^ and 0.862 g/cm^2^, respectively. The medians and quartiles of the participants’ urinary metal concentrations are presented in Table 2, stratified by gender; no difference in the distribution of Mn between men and women, and statistically significant differences were found between men and women for other 10 urinary concentration of metals (*p* < 0.05).

**Table 1:** Characteristics of the study participants in NHANES 2017–2020 (*N* = 1,031).

Characteristic	Total	Men (%)	Women (%)	*p*
*N*	1,031	556 (53.9)	475 (46.1)	–
Age (years, mean ± SE)	64.02 ± 8.798	64.43 ± 8.832	63.53 ± 8.742	0.099
Marital status, *N* (%)				<0.001
Married/Living with partner	623 (60.4)	380 (68.3)	243 (51.2)	
Widowed/Divorced/Separated	324 (31.4)	124 (22.3)	200 (42.1)	
Never married	84 (8.1)	52 (9.4)	32 (6.7)	
Race, *N* (%)				0.877
Mexican-American	93 (9.0)	47 (8.5)	46 (9.7)	
Other Hispanic	111 (10.8)	58 (10.4)	53 (11.2)	
Non-Hispanic White	420 (40.7)	230 (41.4)	190 (40.0)	
Non-Hispanic Black	270 (26.2)	150 (27.0)	120 (25.3)	
Other	137 (13.3)	71 (12.8)	66 (13.9)	
Education, *N* (%)				0.07
Less than 9th grade	80 (7.8)	53 (9.5)	27 (5.7)	
9th–11th grade	112 (10.9)	70 (12.6)	42 (8.8)	
High school graduate or equivalent	278 (27.0)	146 (26.3)	132 (27.8)	
Some college or AA degree	307 (29.8)	149 (26.8)	158 (33.30)	
College graduate or above	254 (24.6)	138 (24.8)	116 (24.4)	
BMI, *N* (%)				0.02
Normal (<25)	248 (24.1)	133 (23.9)	115 (24.20)	
Overweight (25–30)	399 (38.7)	235 (42.3)	164 (34.5)	
Obese (>30)	384 (37.2)	188 (33.8)	196 (41.30)	
Smoking status, *N* (%)				<0.001
Yes	490 (47.5)	328 (59.0)	162 (34.1)	
No	541 (52.5)	228 (41.0)	313 (65.9)	
Intensity of work, *N* (%)				<0.001
Vigorous work	225 (21.9)	148 (26.6)	77 (16.2)	
Moderate work	227 (22.0)	117 (21.0)	110 (23.2)	
No	579 (56.2)	291 (52.3)	288 (60.6)	
Diabetes mellitus, *N* (%)				0.002
Yes	219 (21.2)	138 (24.8)	81 (17.1)	
No	771 (74.8)	391 (70.3)	380 (80.0)	
Borderline	41 (4.0)	27 (4.9)	14 (2.9)	
Hypertension, *N* (%)				0.65
Yes	557 (54.0)	304 (54.7)	253 (53.3)	
No	474 (46.0)	252 (45.3)	222 (46.70)	
Alcohol consumption, *N* (%)				<0.001
Yes	943 (91.5)	528 (95.00)	415 (87.4)	
No	88 (8.5)	28 (5.0)	60 (12.6)	
Household economic status, *N* (%)				
≤1.30	276 (26.8)	153 (27.5)	123 (25.9)	
1.30–1.85	168 (16.3)	94 (16.9)	74 (15.6)	
≥1.85	587 (56.9)	309 (55.6)	278 (58.5)	
BMD (g/cm^2^, mean ± SE)	0.935 ± 0.162	0.998 ± 0.142	0.862 ± 0.152	<0.001

**Table 2:** Medians and quartiles of urinary metal concentrations in NHANES 2017–2020 (*n* = 1,031).

	Total		Men		Women		*P*
Ba	0.978 (0.488, 1.925)		0.826 (0.395, 1.606)		1.171 (0.578, 2.250)		<0.001
Cd	0.340 (0.175, 0.539)		0.243 (0.140, 0.437)		0.412 (0.25, 0.647)		<0.001
Co	0.306 (0.186, 0.489)		0.27 (0.156, 0.433)		0.367 (0.219, 0.567)		<0.001
Cs	4.517 (3.357, 6.382)		4.071 (3.008, 5.524)		5.089 (3.856, 7.162)		<0.001
Mo	35.019 (22.188, 51.758)		32.712 (21.104, 50.307)		38.238 (24.519, 53.351)		0.001
Mn (N)	0 (720)	1 (311)	0 (388)	1 (168)	0 (332)	1 (143)	1.00
Pb	0.409 (0.275, 0.600)		0.371 (0.249, 0.593)		0.440 (0.314, 0.600)		<0.001
Sb	0.044 (0.031, 0.068)		0.041 (0.029, 0.061)		0.048 (0.034, 0.075)		<0.001
Sn	0.582 (0.332, 1.11)		0.472 (0.272, 0.928)		0.733 (0.448, 1.276)		<0.001
Tl	0.108 (0.158, 0.225)		0.14 (0.099, 0.194)		0.183 (0.125, 0.266)		<0.001
Tu	0.054 (0.034, 0.089)		0.049 (0.032, 0.083)		0.060 (0.036, 0.096)		<0.001

### Linear regression and correlation analysis of urinary metals with total BMD

As shown in Table 3, the univariate linear regression analyses revealed that 10 metals other than Mn (*β* = −0.004, 95%CI = −0.0256 – 0.0176) were associated with BMD in total participants, whereas Cs (*β* = −0.0276, 95% CI = −0.0543 − −0.001), Mo (*β* = −0.0287, 95% CI = −0.0504 − −0.007), Sb (*β* = −0.0217, 95% CI = −0.0416 − −0.0018), and W (*β* = −0.0219, 95% CI = −0.0378 − −0.0061) were correlated with femoral BMD only in women. After adjusting for covariates, femoral BMD was negatively and linearly correlated with Ba (*β* = −0.014, 95% CI = −0.0246 − −0.0034), Cd (*β* = −0.0198, 95% CI = −0.0366 − −0.003), Co (*β* = −0.0201, 95% CI = −0.0337 − −0.0064), and Sb (*β* = 0.0167, 95% CI = −0.001 − −0.0324) in men, whereas no metal was found to be linearly correlated with femoral BMD in women. Mn was more than 50% below the LOD, and Mn was excluded to ensure the efficacy of the mixed-exposure test.

**Table 3:** Multiple linear regression analysis of urinary metal concentrations and femoral BMD in NHANES 2017–2020 (*n* = 1,031).

Total	Model 1			Model 2			Model 3		
	β	95% CI	*p*	β	95% CI	*p*	β	95% CI	*p*

Ba	−0.028	(−0.0373, −0.0187)	<0.001	−0.014	(−0.0216, −0.0064)	<0.001	−0.0219	(−0.0306, −0.0133)	<0.001
Cd	−0.0606	(−0.0725, −0.0486)	<0.001	−0.0135	(−0.0244, −0.0025)	0.016	−0.0482	(−0.0602, −0.0363)	<0.001
Co	−0.0442	(−0.0562, −0.0322)	<0.001	−0.0167	(−0.0268, −0.0066)	0.0012	−0.031	(−0.0423, −0.0198)	<0.001
Cs	−0.0431	(−0.0615, −0.0264)	<0.001	0.0056	(−0.01, 0.021)	0.48	−0.0273	(−0.0446, −0.0101)	0.0019
Mo	−0.019	(−0.0336, −0.0045)	0.0103	0.005	(−0.0068, 0.0168)	0.403	−0.0029	(−0.0163, 0.0106)	0.676
Mn	−0.004	(−0.0256, 0.0176)	0.717	−0.0085	(−0.0256, 0.0087)	0.334	−0.0069	(−0.0263, 0.0125)	0.487
Pb	−0.0472	(−0.0618, −0.0327)	<0.001	−0.008	(−0.0213, 0.0034)	0.155	−0.0205	(−0.0348, −0.0001)	0.0047
Sb	−0.0178	(−0.0315, −0.004)	<0.012	0.0034	(−0.0079, 0.0147)	0.553	−0.0095	(−0.0222, 0.0031)	0.14
Sn	−0.0327	(−0.0428, −0.0227)	<0.001	−0.0069	(−0.0155, 0.0017)	0.116	−0.0244	(−0.0339, −0.0149)	<0.001
Tl	−0.0226	(−0.0384, −0.007)	0.005	0.0076	(−0.0056, 0.0208)	0.261	−0.0176	(−0.0323, −0.0029)	0.019
W	−0.0215	(−0.0337, −0.009)	<0.001	−0.0014	(−0.0113, 0.0085)	0.779	0.013	(−0.0242, −0.0019)	0.022
Men									

Ba	−0.0139	(−0.025, −0.0029)	0.013	−0.0147	(−0.025, −0.0045)	0.005	−0.014	(−0.0246, −0.0034)	0.01
Cd	−0.0426	(−0.0571, −0.0281)	<0.001	−0.0211	(−0.0361, −0.0062)	0.006	−0.0198	(−0.0366, −0.003)	0.021
Co	−0.0273	(−0.0416, −0.0132)	<0.001	−0.0167	(−0.0268, −0.0066)	0.001	−0.0201	(−0.0337, −0.0064)	0.004
Cs	0.0028	(−0.0203, 0.0259)	0.457	0.0101	(−0.0117, 0.0319)	0.363	0.0095	(−0.0124, 0.0314)	0.394
Mo	0.0045	(−0.0122, 0.021)	0.599	0.0121	(−0.0036, 0.0278)	0.129	0.0117	(−0.0041, 0.0275)	0.147
Mn	−0.009	(−0.0348, 0.0168)	0.495	−0.0126	(−0.0366, 0.0114)	0.304	−0.0115	(−0.0356,0.0126)	0.348
Pb	−0.0326	(−0.0489, −0.0163)	<0.001	−0.0116	(−0.0276, 0.0044)	0.156	−0.0093	(−0.0259, 0.0073)	0.27
Sb	0.0037	(−0.0125, 0.0199)	0.653	0.017	(0.0016, 0.0326)	0.031	0.0167	(0.001, 0.0324)	0.038
Sn	−0.0149	(−0.0272, −0.0026)	0.018	−0.0089	(−0.0208, 0.0031)	0.388	−0.0103	(−0.0225, 0.0018)	0.096
Tl	0.0134	(−0.0074, 0.0342)	0.205	0.0122	(−0.0073, 0.0317)	0.333	0.0126	(−0.0072, 0.0323)	0.213
W	0.0018	(−0.0141, 0.0177)	0.826	0.0042	(−0.0105, 0.019)	0.572	0.0008	(−0.0141, 0.0158)	0.912
Women									
Ba	−0.0228	(−0.0366, −0.009)	<0.001	−0.0087	(−0.0199, 0.0025)	0.128	−0.0076	(−0.0189, 0.0037)	0.184
Cd	−0.0302	(−0.0496, −0.0109)	0.002	−0.0116	(−0.0275, 0.0043)	0.152	−0.0067	(−0.023, 0.01)	0.429
Co	−0.0349	(−0.0529, −0.0168)	<0.001	−0.0106	(−0.0255, 0.0043)	0.164	−0.0106	(−0.0256, 0.0044)	0.165
Cs	−0.0276	(−0.0543, −0.001)	0.042	0.0037	(−0.0184, 0.0258)	0.74	0.0022	(−0.02, 0.0245)	0.843
Mo	−0.0287	(−0.0504, −0.007)	0.01	−0.0037	(−0.0212, 0.0139)	0.683	−0.0052	(−0.0232, 0.0126)	0.563
Mn	0.0015	(−0.0285, 0.0314)	0.924	−0.0024	(−0.0263, 0.0214)	0.841	−0.002	(−0.0258, 0.0219)	0.872
Pb	−0.0485	(−0.071, −0.0259)	<0.001	−0.006	(−0.0251, 0.0131)	0.537	−0.0003	(−0.02, 0.0195)	0.98
Sb	−0.0217	(−0.0416, −0.0018)	0.0324	−0.0138	(−0.0297, 0.0021)	0.088	−0.0012	(−0.0286, 0.0032)	0.118
Sn	−0.021	(−0.0358, −0.0062)	0.006	−0.006	(−0.018, 0.0061)	0.333	−0.0044	(−0.0167, 0.0078)	0.477
Tl	−0.002	(−0.0235, 0.0194)	0.852	0.0052	(−0.0122, 0.0225)	0.558	0.0039	(−0.0136, 0.0214)	0.661
W	−0.0219	(−0.0378, −0.0061)	0.007	−0.0047	(−0.0177, 0.0082)	0.473	−0.0049	(−0.0178, 0.008)	0.455

Model 1, no covariates were adjusted; Model 2, age, gender, and race were adjusted; Model 3, age, gender, race, education, smoking, drinking, physical activity, BMI, income, diabetes, and hypertension were adjusted.

**Figure 1:**
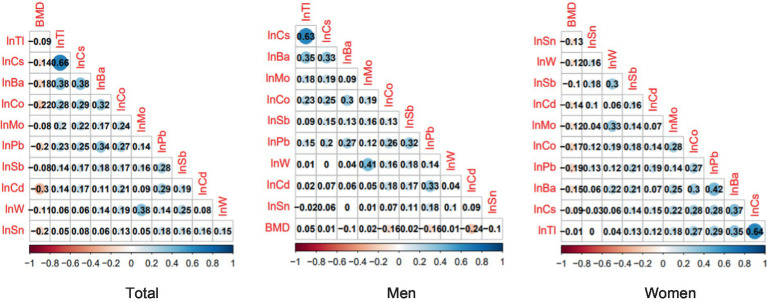
Pearson correlation analysis of urinary concentrations of 10 metals and femoral BMD.

Pearson’s correlation analysis showed a weak negative correlation between all 10 urinary metals and total femoral BMD. Further sex-stratified analyses revealed that Cd (r: −0.24) was more strongly correlated with femoral BMD than others in men, whereas femoral BMD in women had a strongest correlation with Pb (r: −0.19) (Figure 1).

### BKMR analysis

**Figure 2:**
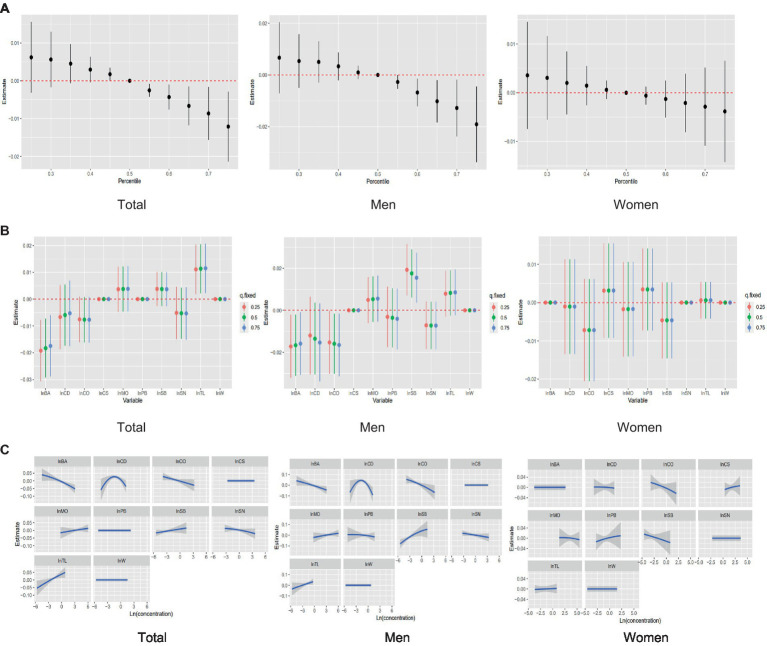
Analysis of the relationship between mixed metal exposure and femoral BMD using the BKMR model. **(A)** The overall effect of a mixture of whole, male, and female individuals on femoral BMD. **(B)** Univariate effects for whole, male, and female. **(C)** Univariate exposure–response relationship for each metal concentration on estimates of femoral BMD.

The combined effect of mixed exposure to the 10 heavy metals on femoral BMD was assessed by fitting a BKMR model. Overall, mixed exposure to the 10 metals was positively correlated with femoral BMD below the 50th percentile, but this effect shifted to a significant negative correlation above the 50th percentile. Gender subgroup analyses showed that mixed metal exposure levels above the 50th percentile had a negative correlation with femoral BMD in men, but a non-significant negative correlation in women (Figure 2A).

The concentrations of the 10 metals were fixed at the 25th, 50th, or 75th percentile to evaluate the effect of each metal on femoral BMD. In the overall analysis, both Ba, Cd and Co were found to have a negative effect on femoral BMD, whereas the effect of Tl was positive. In the sex-stratified analyses, Ba and Co showed negative correlations with femoral BMD, but these correlations were only significant in men. Cd had a stronger negative correlation with femoral BMD in men than in women. Sb was a protective factor in men, being positively correlated with femoral BMD, but was negatively correlated with femoral BMD in women (Figure 2B).

Finally, the non-linear relationship between each metal and femoral BMD was predicted when the concentrations of the other metals were fixed at the 50th percentile. In each subgroup, elevated urinary concentrations of Ba and Co were accompanied by a decrease in femoral BMD; the relationship between Cd concentration and femoral BMD had an inverted U-shape in men. Sb is sex-specific in its correlation with femoral BMD, and a positive correlation is shown in males, while females had a negative correlation (Figure 2C).

### WQS regression analysis

**Figure 3:**
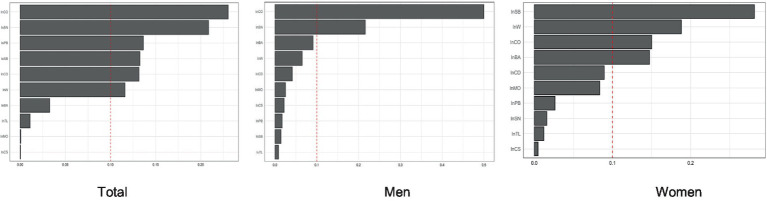
The WSQ regression estimated weights of each of the 11 metals associated with femoral BMD.

WQS regression was performed to analyze the negative effect of the urinary concentrations of the 10 metals on femoral BMD, calculating the percentage weight that each metal accounted for in this effect. The analysis including all participants showed that Co (23.0%) and Sn (20.9%) had the greatest weights of influence on femoral BMD. When the analysis was performed on the gender subgroups, femoral BMD in men was most influenced by Co (50.0%), while the metal with the greatest weight of influence on femoral BMD in women was Sb (28.2%) (Figure 3).

## Discussion

In this study using a nationally representative dataset, various methods with distinct strengths and limitations—multiple linear regression, WQS regression, and BKMR modeling—were combined to assess the effects of urinary metals (Ba, Cd, Co, Cs, Mo, Pb, Sb, Sn, Tl, and W) on total femoral BMD in a middle-aged and older population, with further analysis using gender subgroups. The BKMR model found a significant negative correlation between the concentrations of the 10 urinary metals and femoral BMD in these middle-aged and older adults, including a non-linear relationship of femoral BMD with both Ba and Co. Interestingly, the same trends were obtained in the subgroup of male participants, with the urinary concentrations of Co and Ba accounting for the largest weight in the effect of mixed metal exposure, and Cd also showing a tendency to cause a decrease in BMD, which was not significant. Further analysis of the weighting of the negative effect of urinary metal concentrations on femoral BMD in men showed that Co accounted for the largest proportion of the overall effect among the mixed metals. In women, mixed exposure to the 10 metals showed a trend toward reducing femoral BMD, but no correlations were statistically significant in this analysis. Sb showed a gender difference, being positively correlated with femoral BMD in men but having the opposite effect in women.

The three statistical methods applied in this study yielded consistent results, indicating that Co played a significant role in the reduction of femoral BMD induced by mixed metal exposure, and that the response was more pronounced in male than in female participants. In a previous population-based study, urinary Co concentrations were found to be strongly associated with elevated fasting blood glucose and insulin levels in men, but not in women (23). This suggests that men may be more sensitive to the effects of Co, a toxic metal that has been reported in both eukaryotic and prokaryotic cells to induce the production of reactive oxygen species, superoxide, and free radicals, which damage DNA and inhibit its repair (24). In recent years, Co has been widely used in hip and knee arthroplasty because of its good mechanical properties, which has led to concerns about Co inducing adverse biological reactions such as hypersensitivity and tissue destruction in bone (25, 26). Animal experiments revealed that Co induced lysosomal damage and tissue protease leakage in bone tissue, leading to the activation of the inflammatory vesicle NLRP3, which in turn secreted inflammatory factors such as IL-1β and ultimately induced inflammatory osteolysis (27). In a study of the dose–response relationship between Co and bone homeostasis, *in vivo* toxicity was not observed at Co concentrations of 0.1–5 ppm, although Co was found to cause upregulation of anti-inflammatory, osteogenic, and pro-angiogenic factors at 1 ppm (28). Bone resorption was activated with increasing Co exposure, and an inflammatory response inhibited osteogenic differentiation and caused apoptosis of BMSCs.

The heavy metal Ba is being used in an ever-expanding range of industrial applications; however, it is gradually being discovered that Ba affects metabolic, neurological, and behavioral functions and can cause kidney and cardiovascular disease (29). In general population monometallic exposure studies, urinary Ba concentrations were negatively correlated with total BMD and lumbar spine BMD, and Ba was also found to have a combined effect with Pb and Cd in causing a decrease in BMD, possibly by inducing adverse effects such as oxidative stress (30). In the present study, a non-linear relationship, with an overall trend of negative correlation, between the Ba concentration and femoral BMD was found when the other urinary metals were fixed at median concentrations. Meanwhile, a weak interaction of Ba with other metals was found, with the overall femoral BMD concentrations decreasing as the concentrations of Ba and one other metal increased. Bone tissue is the main target organ for Ba accumulation, which can be attributed to the fact that the radius of Ba ions is similar to that of calcium ions in similar chemical environments (31). With the continuous development of new nanomaterials in recent years, it has been found that Ba-containing nanocomposites can be used as fracture-healing materials, and these have shown beneficial effects on bone tissue healing, such as promotion of bone regeneration and osteoconduction (32). Therefore, the mechanism of Ba-induced BMD reduction is still unclear and needs further examination.

Sb has unique flame retardancy, corrosion resistance, and antioxidant properties. Sb and its compounds are widely used in the production of semiconductors, flame retardants, and pharmaceuticals, and have become emerging environmental pollutants around the world (33). Recently, an increasing number of scientists have paid attention to the relationship between Sb and human health, and whether Sb is harmful to human health is controversial. Chronic exposure to diantimony trioxide impairs particle clearance and promotes the development of lung tumors, adrenal tumors, and lymphomas, as demonstrated by rodent experiments (34). Elsewhere, *in vitro* experiments revealed that exposure to high levels of Sb causes dose-dependent apoptosis in human bronchial epithelial cells (35). Antimony could damage mitochondria through ROS-dependent oxidative stress pathways, and low-dose antimony (0.8uM) could inhibit the level of mitophagy by directly decreasing the PINK1/Parkin pathway (36). In a metabolism-related study, high levels of Sb were found to cause redox imbalance and promote bone loss (37). It might be a promising agent to treat some cancers in an appropriate dose. The polyoxometalate SbW9 inhibits proliferation and induces apoptosis in non-small cell lung cancer cells via the PTEN-dependent AKT signalling pathway (38). In the present study, the Sb concentration was found to affect femoral BMD in a sex-specific manner, being positively correlated with femoral BMD in men and negatively correlated with femoral BMD in women. However, there are few studies on the association between Sb and bone damage, and further *in vivo* and *in vitro* experiments are needed to provide evidential support for the association between Sb and BMD.

The bone mass of an individual peaks in early adulthood and then decreases with age (39). Bone mass is higher in men than women throughout this process, which is consistent with the finding of this study that male femoral BMD was higher than that of women. Estrogen plays an important role in the maintenance of bone homeostasis by promoting anti-apoptosis mechanisms in osteoblasts and osteoclasts and pro-apoptosis mechanisms in osteoclasts (40). Numerous epidemiological studies have found that bone mass in women is significantly affected by estrogen, and that starting around the age of 50 years, women experience an accelerated rate of bone loss due to estrogen deficiency (41). As a result, there are guidelines that recommend screening for osteoporosis using clinical risk assessment tools for menopausal women younger than 65 years, and a variety of preventive and therapeutic medications have been approved by the U.S. Food and Drug Administration (42). The female group in this study, with a mean age of 64 years, were likely to already be receiving anti-osteoporosis treatments. This effective protection may be the reason why no significant effect of mixed metal exposure on femoral BMD was found in women. Alternatively, this absence of a significant correlation may be due to the fact that the effect of estrogen deficiency on BMD greatly outweighs the effect of metal exposure (43).

The present study has the following strengths. First, the data were obtained from the US NHANES database, which is nationally representative. Urinary metals were chosen in this study to reflect bodily metal-exposure levels, and it is well known that urinary concentrations can be a better index of long-term exposure to specific metals than blood concentrations. Second, this study used the BKMR model to investigate the overall trend of the effects of the 10 urinary metals on femoral BMD, as well as WQS regression to further explore the contribution of each metal to the effect of mixed exposure and to avoid bias caused by the covariance of two or more metals. Besides the common heavy metals Cd and Pb, which are linked with an increased risk of osteoporosis, Co, Ba, and Sb were found to affect the femoral BMD in the participants in this study, indicating the need for exposure assessment of these metals. However, the study also has some limitations. The data collected in the most recent round of the NHANES cross-sectional survey were analyzed in a comprehensive manner in this study, but due to the potential influences of drug use, lifestyle behaviors, and government interventions on BMD, more data and experiments are needed to further support the results. Additionally, this study was cross-sectional, making causal inferences difficult and necessitating further support from cohort studies and mixed metal exposure experiments. Finally, a 24-h urine sample does provide a better assessment of an individual’s level of environmental exposure than spot urine sample.

In conclusion, BKMR modeling and WQS regression were applied to investigate the combined effects of multiple metals on femoral BMD in middle-aged and older U.S. citizens. All 10 metals investigated in this study were correlated with reduced femoral BMD, and there were differences between the effects in men and women. Urinary Co, Ba, and Sb appear to play the most important roles in the association of femoral BMD with the combined effects of these metals. Further confirmation of our findings is required in cohort studies and mechanistic experiments.

## Data availability statement

The datasets presented in this study can be found in online repositories. The names of the repository/repositories and accession number(s) can be found in the article/Supplementary material.

## Ethics statement

The studies involving humans were approved by National Center for Health Statistics Institutional Review Board. The studies were conducted in accordance with the local legislation and institutional requirements. The participants provided their written informed consent to participate in this study.

## Author contributions

HL: Writing – original draft, Conceptualization, Data curation, Formal analysis, Resources. GL: Writing – review & editing, Conceptualization. MY: Data curation, Formal analysis, Resources, Writing – review & editing. JZ: Writing – review & editing, Methodology. YD: Methodology, Writing – review & editing. YH: Data curation, Resources, Writing – review & editing. SH: Data curation, Resources, Writing – review & editing. XM: Supervision, Writing – review & editing. LL: Project administration, Supervision, Writing – review & editing.
