# Supplementary material for: Sex-specific associations of urinary mixed-metal concentrations with femoral bone mineral density among older people: an NHANES (2017–2020) analysis
Source: Front Public Health. 2024 May 17;12:1363362. doi: 10.3389/fpubh.2024.1363362 (PMC11140033; doi:10.3389/fpubh.2024.1363362)
Supplement: Supplementary file 1 [file Data_Sheet_1.docx]

| Variable Name | LOD | <LOD(%) |
| --- | --- | --- |
| **Ba** | 0.084 | 1.16 |
| **Cd** | 0.055 | 5.53 |
| **Cs** | 0.13 | 0.00 |
| **Co** | 0.024 | 1.07 |
| **Mn** | 0.13 | 69.84 |
| **Mo** | 0.8 | 0.00 |
| **Pb** | 0.03 | 0.19 |
| **Sb** | 0.022 | 23.38 |
| **Tl** | 0.018 | 0.68 |
| **Sn** | 0.2 | 17.17 |
| **W** | 0.018 | 16.49 |

supplementary table 1. LOD for metals and total population detection rate for each metal.
